# Effect of the preoperative prognostic nutritional index on the long-term prognosis in patients with borderline resectable pancreatic cancer after pancreaticoduodenectomy

**DOI:** 10.3389/fonc.2023.1098459

**Published:** 2023-05-01

**Authors:** Jin-Can Huang, Bing Pan, Tao Jiang, Xin-Xue Zhang, Shao-Cheng Lyu, Ren Lang

**Affiliations:** Department of Hepatobiliary Surgery, Beijing Chao-Yang Hospital Capital Medical University, Beijing, China

**Keywords:** borderline resectable pancreatic cancer, preoperative prognostic nutritional index, prognostic factor, tumor recurrence, risk factor

## Abstract

**Background:**

The preoperative prognostic nutritional index (PNI) is an indicator of systemic immune-nutritional condition and is a well-known prognostic biomarker in cancer patients. This study aims to reflect the correlation between the preoperative PNI and prognosis in patients with borderline resectable pancreatic cancer (BRPC) after pancreaticoduodenectomy (PD).

**Methods:**

Medical records of patients with BRPC after PD between Jan 2011 and Dec 2021 in our hospital were retrospectively analyzed. The preoperative PNI was calculated, and the receiver operating characteristic curve was obtained based on the preoperative PNI and the 1-year survival rate. Patients were divided into two groups (High-PNI and Low-PNI) following the best cut-off value of the preoperative PNI, and demographic and pathologic findings were compared between the two groups. Univariate and multivariate analysis were performed to identify risk factors in recurrence and long-term survival.

**Results:**

The best cut-off value for the preoperative PNI was 44.6 (sensitivity: 62.46%; specificity: 83.33%; area under the curve: 0.724). Patients in the low-PNI group had significantly shorter recurrence-free survival (P=0.008) and overall survival (P=0.009). The preoperative PNI (P=0.009) and lymph node metastasis (P=0.04) were independent risk factors for tumor recurrence. The preoperative PNI (P=0.001), lymph node metastasis (P=0.04), neoadjuvant chemotherapy (P=0.04) were independent risk factors for long-term survival in patients.

**Conclusion:**

The preoperative PNI, lymph node metastasis, neoadjuvant chemotherapy were independent risk factors for recurrence and long-term survival in patients with BRPC. The preoperative PNI might be an indicator that can predict BRPC patients’ recurrence and survival. Patients with high-PNI would benefit from neoadjuvant chemotherapy.

## Introduction

Pancreatic cancer (PC) is an intractable malignancy and is the 7th leading cause of global cancer (1). Compared with many other cancers, the combined five-year survival rate for PC is very low at just 5% to 10%. This is because early-stage pancreatic cancer is usually clinically silent, and most people who present with symptoms attributable to pancreatic cancer have advanced diseases. In 2006, Varadhachary et al. (2) proposed criteria for borderline resectable pancreatic cancer (BRPC) for the first time, defining which kind of BRPC patients with vascular invasion was suitable for surgical treatment. Concerning the treatment of BRPC, pancreaticoduodenectomy (PD) combined with venous resection and reconstruction has been widely acknowledged (3, 4). Meanwhile, some consensus guidelines recommended neoadjuvant chemotherapy for patients with BRPC (5, 6). Although advances in diagnostic approaches, perioperative management, radiotherapy techniques, and systemic therapies for BRPC have been made, surgical outcomes and overall survival rates of patients with BRPC are still bleak. Therefore, the importance of predicting the long-term prognosis for patients with PC before surgery is emphasized to make the best treatment decision.

As a vital digestive organ, the pancreas plays a significant role in digestion and glycemic control. Up to 80% of PC patients suffer from malnutrition as a result of pancreatic exocrine and endocrine insufficiency (7). Besides, as one of the most common symptoms occurs in PC patients, jaundice may further aggravate the nutritional status by reducing fat digestion and fat-soluble vitamin absorption. The prognostic nutritional index (PNI) is a measure of both the nutritional and systemic immunological condition of a patient which is calculated based on serum albumin concentrations and the total lymphocyte count in peripheral blood, and was originally proposed to assess perioperative immunonutritional status (8). Recently, the PNI was shown to be a prognostic marker for various malignancies (9–11), and especially the preoperative PNI was an independent postoperative prognostic factor even in lung cancer patients (12, 13). Nevertheless, few studies have objectively evaluated the relationship between the preoperative PNI and prognosis in patients with BRPC. This study aimed to retrospectively analyze and reflect the correlation between the preoperative PNI and prognosis in patients with BRPC after PD.

## Materials and methods

### Patient selection

Between Jan 2011 and Dec 2021, a total of 122 patients who received PD for histologically proven BRPC were enrolled in this study. The criteria for BRPC were defined by preoperative resectability status of the National Comprehensive Cancer Network (NCCN) guidelines Version 2.2021 (14). Patients were divided into two groups (High-PNI and Low-PNI) following the best cut-off value of the preoperative PNI. The study was conducted in accordance with the Declaration of Helsinki (as revised in 2013) and was approved by the Ethics Committee of Beijing Chao-Yang Hospital (No. 2020-D-301). All patients and families were informed and agreed with the therapy strategies in this study. The need for consent was waived because of the retrospective nature of the study and the analysis used anonymous clinical data, which was approved by the Ethics Committee of Beijing Chaoyang Hospital. Furthermore, we informed all patients or their families about our study, and obtained informed consent from patients or their families during the follow-up.

### Data collection

Demographic information, preoperative medical data, intraoperative condition, and histology results were collected. Postoperative follow-ups consisted of chest and abdominal computed tomography (CT), and bone scintigraphy at 6-month intervals during the 1st year and yearly thereafter. Blood tests (routine complete blood count, blood chemistry and enzyme test) and tumor markers were checked at 3- or 4-month intervals during the first year and at 6-month intervals thereafter. The first appearance of any new lesion suspected to be recurrence of the original PC was defined as postoperative recurrence, and was clinically diagnosed by combinations of CT, MRI, a bone scintigram, or was pathologically diagnosed if necessary. The postoperative recurrence-free survival (RFS) was defined as the interval between the operation and the first recurrence event. The postoperative overall survival (OS) was calculated from the time of operation to the date of death from any cause.

### Calculation of the preoperative PNI and the cut-off value

The preoperative PNI was calculated using the following formula: serum albumin levels (g/L) + 5×total lymphocyte count (10^9^/L) in peripheral blood. (15). The values for total lymphocytes and albumin were obtained within 7 days preoperatively. The receiver operating characteristic curve was obtained based on the preoperative PNI and the 1-year survival rate, from which the best cut-off value of PNI was determined to be 44.6 (sensitivity: 62.46%; specificity: 83.33%; area under the curve: 0.724). Fifty-seven (46.72%) patients had preoperative PNI >44.6 (high-PNI) and the remaining 65 (53.28%) patients had a lower preoperative PNI (low-PNI).

### Statistical analysis

Continuous variables conformed to the normal distribution were expressed as mean and standard deviation and compared using the Student’s t-test. The non-normal distribution was expressed as median with interquartile range (IQR). Categorical variables were analysed using Fisher’s exact test. Patients’ survival was analysed by using the Kaplan–Meier method and compared groups using the log-rank test. The Cox proportional hazards model was applied to identify independent prognostic factors. P<0.05 was considered significant. All statistical analyses were performed using SPSS, version 22.0 (IBM).

## Results

### Association between patients’ characteristics and the preoperative PNI

The preoperative low-PNI was significantly associated with postoperative tumor recurrence (P<0.001), and but no other factors, including gender, age, preoperative leucocytes, albumin, carbohydrate antigen 19-9 (CA19-9), preoperative drainage for jaundice, child-pugh grade, history of diabetes, neoadjuvant chemotherapy, intraoperative blood loss volume, blood transfusion, operative time, tumour size and differentiation, resection margin, lymph node metastasis, postoperative complications and postoperative hospital stay (**Table 1**).

**Table 1:** Demographic and pathologic findings in two groups.

Variables	High-PNI (n=57)	Low-PNI (n=65)	P-value
Gender (Male/Female)	25/32	37/28	0.15
Age (Year)	65.6±9.1	59.0±10.3	0.55
Preoperative leucocytes(×10^9^ /L)	1.1±0.5	1.7±0.5	0.56
Preoperative albumin (g/L)	33.7±3.1	40.7±4.1	0.62
Preoperative CA19-9 (U/ml)	204.0(32.7,735.4)	175.5(30.8,693.1)	0.91
Preoperative drainage for jaundice (Yes/No)	11/46	14/51	0.76
Child-Pugh grade(A/B)	44/13	51/14	0.87
History of diabetes (Yes/No)	19/38	20/45	0.76
neoadjuvant chemotherapy (Yes/No)	9/48	13/52	0.54
Intraoperative blood loss (ml)	500 (400,950)	600 (400,1000)	0.70
Blood transfusion (Yes/No)	27/30	37/28	0.29
Operative time (h)	12.0 (10.0, 14.7)	12.0 (11.0, 14.0)	0.57
Tumour size (cm)	3.7 (3.0, 5.0)	3.5 (3.0, 4.3)	0.07
Tumour differentiation (poor/ moderate - high)	22/35	22/43	0.58
Pancreatic resection margin (R0/R1)	53/4	60/5	0.88
Lymph node metastasis (Yes/No)	41/16	42/23	0.38
Postoperative complications (Yes/No)	16/41	20/45	0.74
Postoperative hospital stays (d)	21.0 (15.0, 32.0)	23.0 (16.3, 38.3)	0.22
Postoperative chemotherapy (Yes/No)	31/26	18/47	0.003
Postoperative tumor recurrence (Yes/No)	12/45	40/25	<0.001

### Prognostic factors in patients with BRPC after PD

We compared RFS and OS in patients who were older versus younger than 60 years, males *vs*. females, diabetes *vs*. no-diabetes, preoperative drainage for jaundice (yes *vs*. no), preoperative serum CA19-9 levels (abnormal *vs*. normal), preoperative serum albumin levels (abnormal *vs*. normal), preoperative total lymphocyte count (≤1 *vs*.>1×10^9^/L), the preoperative PNI (low *vs*. high), neoadjuvant chemotherapy pathological (yes *vs*. no), operative time (≤10 *vs*.>10 h), intraoperative blood loss (≤500 *vs*.>500 ml), intraoperative blood transfusion (yes *vs*. no), tumour differentiation (poor *vs*. moderate-high), tumour size (≤2 *vs*.>2cm), pancreatic resection margin (R0 *vs*. R1), lymph node metastasis (yes *vs*. no), postoperative chemotherapy (yes *vs*. no). Univariate analyses (**Table 2**) showed that age (P=0.04), the preoperative PNI (P=0.008), neoadjuvant chemotherapy (P=0.02), lymph node metastasis (P=0.04), significantly affected RFS. The neoadjuvant chemotherapy regimens included: fluorouracil, leucovorin, irinotecan, and oxaliplatin or gemcitabine plus nanoparticle albumin-bound (nab)-paclitaxel. The relative risks (RRs) for patients with age younger than 60 were 0.802 versus patients with age older than 60 (95%CI: 0.519-1.238), 1.981 for low-PNI patients versus high-PNI patients (95%CI: 1.278-3.070), neoadjuvant chemotherapy (RR:0.707; 95%CI:0.370-1.353), lymph node metastasis (RR:1.159; 95%CI:0.729-1.843). In multivariate analysis, the preoperative PNI (RR: 1.765; 95%CI: 1.156-2.696; P=0.009), lymph node metastasis (RR:1.58; 95%CI:1.009-2.475; P=0.04) were independent prognostic factors (**Table 3**) in RFS.

**Table 2:** Univariate analysis of risk factors for BRCP recurrence.

Variables	Number (n=122)	1-year survival rate (%)	2-year survival rate (%)	χ2	P-value
Gender Male Female	62 60	45.3 56.1	25.8 41.6	3.111	0.07
Age (Year) ≤60 >60	51 71	64.8 41.3	46.9 24.1	3.951	0.04
Diabetes Yes No	39 83	52.0 57.1	30.1 35.0	0.058	0.81
Drainage for jaundice Yes No	25 9	60.0 48.2	45.3 30.0	2.249	0.13
Serum Albumin (g/L) ≤35 >35	42 80	35.7 59.3	23.0 39.5	3.109	0.07
Total lymphocyte count(×10^9^/L) ≤1 >1	24 98	33.8 54.9	28.1 34.8	3.696	0.05
Preoperative PNI ≤44.6 >44.6	57 65	34.0 66.2	20.8 45.6	7.006	0.008
Preoperative CA19-9 (U/ml) ≤37 >37	33 89	53.2 44.7	32.6 22.9	1.403	0.23
neoadjuvant chemotherapy Yes No	22 100	74.8 45.5	61.8 27.5	5.058	0.02
Operative time (h) ≤10 >10	30 92	54.7 40.0	40.4 20.0	3.293	0.07
Intraoperative blood loss (ml) ≤500 >500	57 65	52.1 49.9	35.1 32	0.003	0.95
Blood transfusion Yes No	64 58	44.7 54.2	33.6 33.7	0.000	0.98
Tumour differentiation Poor Moderate - high	44 78	50.3 51.9	31.7 34.2	0.024	0.87
Tumour size (cm) ≤2 >2	14 108	51.1 48.2	36.2 33.0	0.085	0.77
Pancreatic resection margin R0 R1	113 9	53.2 22.2	34.2 22.2	0.958	0.32
Lymph node metastasis Yes No	83 39	46.2 53.3	12.4 45.3	4.149	0.04
Postoperative chemotherapy Yes No	49 73	52.7 48.4	34.2 33.0	0.045	0.83

**Table 3:** Multivariate analysis of independent risk factors for BRPC recurrence.

Variables	RR value	95% CI	P-value
Age	1.198	0.773 – 1.858	0.41
PNI	1.765	1.156 - 2.696	0.009
neoadjuvant chemotherapy	1.810	0.997 - 3.286	0.05
Lymph node metastasis	1.580	1.009 - 2.475	0.04

Univariate analysis (**Table 4**) showed that age (P=0.04), the preoperative PNI (P=0.009), neoadjuvant chemotherapy (P=0.02), lymph node metastasis (P=0.04) significantly affected OS. The RR for patients with age younger than 60 was 0.840 versus patients with age older than 60 (95%CI: 0.677-1.042), 1.270 for low-PNI patients versus high-PNI patients (95%CI: 1.042-1.548), neoadjuvant chemotherapy (RR: 0.739; 95%CI: 0.515-1.060), lymph node metastasis (RR: 0.779, 95%CI: 0.653-0.929). In multivariate analysis, the preoperative PNI (RR: 1.748, 95%CI: 1.145-2.668, P=0.01), neoadjuvant chemotherapy (RR: 1.835; 95%CI: 1.011-3.331; P=0.04), lymph node metastasis (RR: 1.575; 95%CI: 1.006-2.465; P=0.04), were independent prognostic factors (**Table 5**) in OS.

**Table 4:** Univariate analysis of long-term survival in patients with BRCP.

Variables	Number (n=122)	1-year survival rate (%)	2-year survival rate (%)	*χ* ^2^	P-value
Gender Male Female	62 60	52.8 59.4	25.8 41.1	3.167	0.07
Age (Year) ≤60 >60	51 71	71.5 45.8	46.3 24.3	4.149	0.04
Diabetes Yes No	39 83	54.2 57.0	30.1 34.4	0.138	0.71
Drainage for jaundice Yes No	25 97	64.0 53.9	44.8 29.7	2.471	0.11
Serum Albumin (g/L) ≤35 >35	42 80	42.9 63.4	22.7 39.2	3.113	0.07
Total lymphocyte count(×10^9^/L) ≤1 >1	24 98	34.8 61.3	29.0 34.4	3.752	0.05
PNI ≤44.6 >44.6	57 65	41.3 69.5	20.6 45.5	6.814	0.009
Preoperative CA19-9 (U/ml) ≤37 >37	33 89	56.7 54.1	37.1 23.0	1.266	0.26
neoadjuvant chemotherapy Yes No	22 100	86.4 49.6	61.8 27.2	5.199	0.02
Operative time (h) ≤10 >10	30 92	58.2 56.7	38.4 20.0	3.265	0.07
Intraoperative blood loss (ml) ≤500 >500	57 65	57.1 55.5	34.6 31.9	0.006	0.93
Blood transfusion Yes No	64 58	54.7 57.8	33.0 33.4	0.033	0.85
Tumour differentiation Poor Moderate-high	44 78	55.3 58.2	32.0 33.7	0.009	0.92
Tumour size (cm) ≤2 >2	14 108	57.1 47.1	35.4 32.8	0.088	0.76
Pancreatic resection margin R0 R1	113 9	59.0 22.2	33.9 22.2	0.961	0.32
Lymph node metastasis Yes No	83 39	51.3 59.0	12.3 44.9	4.122	0.04
Postoperative chemotherapy Yes No	49 73	56.9 55.5	33.4 32.9	0.156	0.69

**Table 5:** Multivariate analysis of long-term survival in patients with BRCP.

Variables	*RR* value	95% *CI*	P-value
Age	1.222	0.789 - 1.893	0.36
PNI	1.748	1.145 - 2.668	0.01
neoadjuvant chemotherapy	1.835	1.011 - 3.331	0.04
Lymph node metastasis	1.575	1.006 - 2.465	0.04

### Correlation between the preoperative PNI and RFS, OS in patients with BRPC after PD

**Figure 1:**
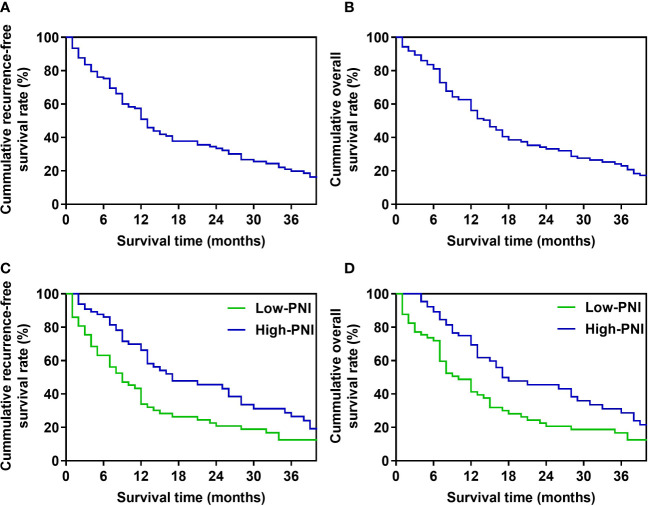
Survival curve of patients **(A)**. Recurrence-free survival curve of the whole study’s patients. **(B)**. Overall survival curve of the whole study’s patients. **(C)**. Recurrence-free survival curve of high-PNI and low-PNI groups. **(D)**. Overall survival curve of high-PNI and low-PNI groups.

For patients in the whole study, the median recurrence-free survival (RFS) time was 13 months, and the 1-, 2-, 3-year RFS rates were 50.8%, 33.4%, and 19.8%, respectively (**Figure 1A**). The median OS was 15 months, and the OS rates in 1 year, 2 years, and 3 years after PD were 56.1%, 33.1%, and 23.0%, respectively (**Figure 1B**). In Kaplan-Meier analysis of RFS, and OS by the preoperative PNI, patients in the low-PNI group had a significantly shorter RFS (P=0.008, log-rank test, **Figure 1C**), OS (P=0.009, log-rank test, **Figure 1D**) than the high-PNI group. The median RFS of patients in low-PNI and high-PNI was 9 and 17 months, respectively, and the 1-, 2- and 3-year RFS rates were 34.0%, 20.8%, 12.6%, and 66.2%, 45.6%, 28.8%, respectively. The median OS in the two groups was 10 and 17 months, and the 1-, 2- and 3-year OS rates were 41.3%, 20.6%, 16.7%, and 69.5%, 45.5%, 28.7%, respectively.

## Discussion

Tumor of BRPC invades the portal vein system and is adjacent to the vital celiac arteries. Although the radical PD combined with venous resection and reconstruction has been widely recognized (16, 17), it is a complex operational process with a dismal long-term prognosis. In addition, the patients are often in a state of negative nutritional balance and low immune function when admitted to the hospital, and less than 20% of patients are eligible for surgical resection at the time of presentation (18, 19). A prospective study of 97 patients with pancreatic cancer by Poulia et al. (20) showed that impaired preoperative nutritional status was associated with shorter RFS and OS. Therefore, the preoperative evaluation of the nutritional and immune status of patients with BRPC is very important for the choice of treatment.

Previous studies found that factors such as tumor size, lymph node metastasis, TNM stage, and tumor markers have predictive value for the prognosis of patients with BRPC (21). Still, most of these predictors are challenging to assess the condition of patients before surgery. An increasing number of studies have pointed out the significance of preoperative assessment of patients’ immune and nutritional status in assessing their long-term outcomes after surgery. As early as 1984, Japanese scholars first proposed PNI to evaluate the perioperative condition of patients with gastrointestinal tumors and predict the operation risk by combining albumin and the total number of lymphocytes in peripheral blood. As a simple and effective preoperative immune and nutritional evaluation index, the application of PNI in BRPC still needs more pieces of evidence.

Serum albumin is produced by the liver, and low serum albumin levels reflect the state of nutritional consumption or impaired liver synthesis. Eckart et al. (22) conducted a retrospective analysis of 2465 patients in the emergency department, showed that hypoalbuminemia was significantly correlated with elevated inflammatory index and nutritional risk, and albumin could predict short-term mortality. Many pro-inflammatory cytokines, such as IL-1, IL-6, and TNF- α, are involved in regulating the production of albumin in hepatocytes, and they also play a particular role in regulating angiogenesis and cancer progression (23). Therefore, serum albumin, as a common nutritional index, can also reflect the inflammatory state of the body. The total number of peripheral blood lymphocytes is another component of PNI. As an essential cell subgroup involved in tumor immunity, it antagonizes the cytotoxicity of cancer cells and participates in the proliferation, migration, and invasion of cancer cells (24). Among them, CD4^+^ and CD8^+^ T lymphocytes play a crucial role in improving anti-tumor immunity. CD4^+^ T lymphocytes can initiate or maintain immune response by secreting IL-10 or activating antigen-presenting cells and playing a pivotal role. CD8^+^ T lymphocytes can recognize tumor-associated antigens through MHC-I molecules, thus inhibiting tumor proliferation (25, 26). In addition, studies have shown that lymphocytopenia is associated with poor prognosis in tumor patients (27).

As a combined index of serum albumin and lymphocytes, PNI can effectively improve their predictive sensitivity. Many studies have confirmed its role in predicting the prognosis of digestive tract malignant tumors. Kurahara et al. (28) analyzed the OS of 96 patients with locally advanced PC treated with radiotherapy and chemotherapy, and found that PNI could be an effective index to predict the survival time of patients. In addition, Nozoe et al. (29)studied the relationship between the preoperative PNI and clinicopathological factors in 248 patients with gastric cancer, and suggested that the preoperative low-PNI was significantly correlated with greater depth of tumor invasion, lymph node metastasis, venous invasion, and distant metastasis, and invasiveness. Generally speaking, the PNI reflects individual-related factors such as nutritional status, inflammatory response, and immune function, which were closely related to the progression and prognosis of malignant tumors. In this study, the RFS and OS of patients with low-PNI were significantly shorter than that of patients with high-PNI. So the preoperative PNI, as a nutritional and immune index, can effectively predict the long-term prognosis of patients with BRPC.

Affected by tumor location, malignant degree, and other factors, most patients with PC will develop malnutrition-related symptoms, mainly unconscious weight loss as the first manifestation (30). Although there are many nutrition assessment methods, including nutrition risk screening 2002 and malnutrition universal screening tool, which are widely used in patients’ nutrition assessment, the results are affected mainly by the subjective description and judgment from patients and evaluators. They cannot effectively predict the prognosis of patients (31). As an objective formula based on laboratory examination, the PNI has excellent potential in the preoperative evaluation of BRPC. Since 2014, NCCN guidelines have recommended neoadjuvant chemotherapy for BRPC patients, in addition to immediate surgery. Studies have shown that neoadjuvant therapy can inhibit tumor progression and effectively improve postoperative survival time (32). However, there is still a lack of unified criteria for screening patients suitable for neoadjuvant therapy. Univariate and multivariate analysis in this study showed that the prognosis of patients with high-PNI and neoadjuvant chemotherapy is better, so using the preoperative PNI to evaluate the prognosis of patients after operation also provides sufficient evidence for neoadjuvant therapy and long-term benefits for patients.

The main limitation of this study is that it represented a single center’s experience. The number of patients in each group is relatively small, which may limit the accuracy of our assessment. We will further expand the sample size of the enrolled patients in a multicenter to confirm the findings and find more stable and cogent conclusions.

## Conclusions

To sum up, the preoperative PNI, lymph node metastasis, neoadjuvant chemotherapy were independent risk factors for recurrence and long-term survival in patients with BRPC. The preoperative PNI might be an indicator that can predict BRPC patients’ prognosis, and patients with low-PNI would have a dismal prognosis. However, the prognosis of patients with high-PNI and neoadjuvant chemotherapy is better. At present, multicenter, extensive sample data are still needed to accurately evaluate the prognosis of patients with BRPC before the operation to provide more theoretical support for precise treatment.

## Data availability statement

The original contributions presented in the study are included in the article/supplementary material. Further inquiries can be directed to the corresponding authors.

## Ethics statement

The study was conducted in accordance with the Declaration of Helsinki (as revised in 2013) and was approved by the Ethics Committee of Beijing Chao-Yang Hospital (No. 2020-D-301). All patients and families were informed and agreed with the therapy strategies in this study. The need for consent was waived because of the retrospective nature of the study and the analysis used anonymous clinical data, which was approved by the Ethics Committee of Beijing Chao-Yang Hospital. Furthermore, we informed all patients or their families about our study, and obtained informed consent from patients or their families during the follow-up. The ethics committee waived the requirement of written informed consent for participation.

## Author contributions

Conception and design: RL, S-CL, J-CH, BP. Administrative support: RL, S-CL. Provision of study materials or patients: J-CH, BP. Collection and assembly of data: J-CH, BP, TJ, X-XZ. Data analysis and interpretation: J-CH, BP. Manuscript writing: J-CH, BP. All authors read and approved the final version of the manuscript. J-CH and BP contribute equally to this manuscript and share the first authorship. All authors contributed to the article and approved the submitted version.
